# Blend Segregation in Tablets Manufacturing and Its Effect on Drug Content Uniformity—A Review

**DOI:** 10.3390/pharmaceutics13111909

**Published:** 2021-11-11

**Authors:** Emilia Jakubowska, Natalia Ciepluch

**Affiliations:** 1 Chair and Department of Pharmaceutical Technology, Faculty of Pharmacy, Poznan University of Medical Sciences, 6 Grunwaldzka Street, 60-780 Poznan, Poland; 2 Department of Medical Rescue, Chair of Emergency Medicine, Faculty of Health Sciences, Poznan University of Medical Sciences, 7 Rokietnicka Street, 60-806 Poznan, Poland; nbaranowska@ump.edu.pl

**Keywords:** blend segregation, powder segregation, content uniformity, blend uniformity, blend homogeneity, tablet manufacturing

## Abstract

Content uniformity (CU) of the active pharmaceutical ingredient is a critical quality attribute of tablets as a dosage form, ensuring reproducible drug potency. Failure to meet the accepted uniformity in the final product may be caused either by suboptimal mixing and insufficient initial blend homogeneity, or may result from further particle segregation during storage, transfer or the compression process itself. This review presents the most relevant powder segregation mechanisms in tablet manufacturing and summarizes the currently available, up-to-date research on segregation and uniformity loss at the various stages of production process—the blend transfer from the bulk container to the tablet press, filling and discharge from the feeding hopper, as well as die filling. Formulation and processing factors affecting the occurrence of segregation and tablets’ CU are reviewed and recommendations for minimizing the risk of content uniformity failure in tablets are considered herein, including the perspective of continuous manufacturing.

## 1. Introduction

The safety and efficacy of solid oral drug products, as well as their robust performance, are ensured by meeting the specified values of critical quality attributes (CQAs). Among these, content uniformity (CU) in drug products is of primary importance. A repeatable unit content of the active pharmaceutical ingredient (API) and a drug potency remaining within the acceptable deviation from the target value (as a general simplification, 85–115% of the label claim for most products) are fundamental for assuring a therapeutic concentration in systemic circulation and for minimizing the risk of adverse events. This requirement is particularly important in the case of low-dose, highly potent drugs, where small variations in the API amount due to the dosage form may result in a significant impact on safety and efficacy.

In routine quality control of solid dosage form manufacturing, CU testing is conducted according to the harmonized rules described in the USP (chapter 905) and Ph. Eur. (chapter 2.9.40). Nevertheless, the best practice for evaluating the homogeneity of both blends (as intermediates) and final products, including optimal sampling methods, statistical analysis and acceptability criteria, is the topic of ongoing discussion, as demonstrated, e.g., by the withdrawal of FDA draft guidance in 2013 [1,2]. A detailed review of different approaches and recommendations for evaluation procedures can be found in specific publications, e.g., [3,4,5], and as such these are outside the scope of this review. 

Regardless of the CU assessment according to basic Quality-by-Testing approach, current drug product development and manufacturing should align with Quality-by-Design principles, where formulation and processing parameters are thoroughly understood with respect to their impact on product CQAs. Under this paradigm, mechanisms leading to possible quality failure should be explored and mitigated. Regarding the content uniformity of tablets, which are the most popular solid dosage form, it is a common belief that issues with homogeneity especially concern tablets manufactured by means of direct compression, as opposed to employing wet or dry granulation methods, of which the aim is, among others, to improve tableting blend homogeneity. Industry surveys, however, do not confirm this view, indicating that sufficient drug potency and content is routinely achieved [6]. Nevertheless, unacceptable variability in the API amount or tablet sub-/superpotency might be encountered and can stem from different sources. Prescott and Garcia have reviewed several patterns of changes in the API content and its relative standard deviation (RSD) during the time of blending or compression, such as high within-location or between-location variability, stray values and trending. The observation of a particular pattern of CU deviation can help to identify the technological or analytical causes of the problem, the most common of which are: improper particle distribution (e.g., agglomeration); poor macro- and microblending at the powder mixing stage; loss of a component (e.g., due to adsorption to the equipment surface); thief sampling and analytical errors; and finally, segregation of well-mixed blends during powder transfer, handling or further operations [7].

Therefore in general, two processing causes of failed CU may be broadly identified: suboptimal mixing of ingredients and the failure to meet required blend uniformity as an intermediate, or segregation of initially well-mixed material during handling or compression. This review focuses on the latter phenomenon, i.e., demixing and the resulting inhomogeneous distribution of ingredients during various stages of tablet manufacturing, whereas the topic of blending as a unit operation is outside its scope. Basic mechanisms or types of powder segregation are covered and testing methods are briefly mentioned. The primary focus of this paper is a comprehensive review of research on blend segregation phenomena related to solid dosage form manufacturing, both as real industrial case studies and as simulation studies of which the settings and results can be considered relevant to pharmaceutical context. Although there exist several review papers on segregation [7,8,9], to the authors’ knowledge an elementary yet exhaustive summary of recent research on demixing in pharmaceutical manufacturing is missing. Therefore, the current work aims to fill this gap, focusing on the factors affecting particle segregation and the resulting loss of content uniformity, as well as their mitigation at different stages or operations in tablet production. According to this, the review covers such areas as the transfer of blends, discharge from bins or hoppers and, finally, die filling during the compression process in the context of factors influencing powder segregation events and issues with tablets’ content uniformity. Research on the formulation and granulation parameters related to the topic is also considered. Moreover, a perspective on continuous manufacturing as a means to reduce segregation and improve content uniformity is also reviewed and highlighted. The review methodology is presented in Appendix A. A caveat must be made that the diverse reviewed studies use varied measures for assessing CU in terms of responses (e.g., RSD of API content, acceptance value (AV) according to pharmacopeia, etc.) and different, often custom ways of calculating segregation indices. Due to this disparity, a direct comparison between the reports in terms of absolute results is not warranted. It is worth noting that the situation has recently been recognized and recommendations for streamlining the mixing/segregation measures are being made [10]. Here, focus is placed on the observed trends and relationships between processing parameters, segregation phenomena and content uniformity changes.

## 2. Powder Segregation Basics

### 2.1. Segregation Mechanisms and Causes

**Figure 1:**
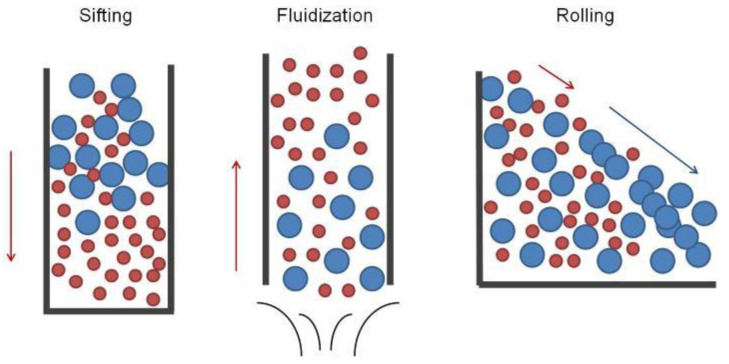
A schematic representation of main particle segregation mechanisms.

Although as many as 13 mechanisms of the segregation of ingredients have been identified in various engineering areas [11], in pharmaceutical solids handling, only a few are relevant: sifting, fluidization (entrainment of air) or entrainment of particles in airstream and rolling segregation (Figure 1) [9,12].

Sifting (sieving, percolation) is the most common mechanism of component separation. In this phenomenon, particles of smaller size move downwards due to the gravitational force through the void spaces in the bed of larger particles. As a result, components are unevenly distributed throughout the powder mass, with the larger particles forming the top layer. For sifting to occur, several conditions must be fulfilled, i.e.,

- the particle size ratio of segregating components in a binary mixture must be at least 1.3:1
- sufficiently large mean particle size; the exact universal value, however, has not been determined
- free-flowing material
- velocity gradient between moving particles [8,13].

Apart from a material’s properties, the occurrence of sifting depends on the geometry of the equipment and the type of flow related to it. Essentially, the flow during the discharge from a bin or a hopper can be either characterized as mass flow or funnel flow. Funnel flow, an undesirable phenomenon leading to particle segregation, takes place in hoppers of which the walls are too shallow or rough for easy particle sliding. The powder residing close to the walls is stagnant, whereas the remaining particles flow through a central, funnel-shaped channel, which results in the ‘first in, last out’ type of particle movement. This problem does not exist in the case of mass flow (‘first in, first out’), in which the entire mass is uniformly discharged at the same time. The risk of particle segregation due to funnel flow can be eliminated by ensuring mass flow through proper equipment design, such as choosing conical instead of pyramidal hoppers or using inserts [12].

Fluidization and entrainment in air is another segregation mechanism depending on the difference in the particle size of components. Fine particles have lower air permeability than coarse ones, which causes them to retain air in void spaces for a longer time and consequently to be deposited on the top of the surface after discharge or during the filling of containers. Similarly, fine particles are more sensitive to counter-currents of air, and air drag’s effects on them are more significant than those for larger particles. This results in their lower free-fall velocity and consequently in the deposition at the top of the falling powder bed, as well as in their unpredicted scattering, deviating from calculated trajectories [12]. Therefore, the effect of air-induced segregation on blend inhomogeneity is opposite to that of sifting, with finer particles forming the top layer of the powder bed.

Another demixing mechanism is rolling, which can be considered a more narrow subtype of trajectory segregation, where particles of different sizes gain different velocities due to resistance forces opposing their momentum at different magnitudes [9,14]. As a result, disparate size fractions are distributed over different locations. In the specific case of rolling segregation, large particles slide over the powder heap surface faster than fine particles, depositing at the bed’s bottom and outer areas (Figure 1).

The difference in particle size of the mixture ingredients is a key condition for powder segregation, regardless of the mechanism. Interestingly, the significance of the polydispersity or size distribution width has been rather scarcely explored. On the one hand, as Tang and Puri point out, older reports suggest that it is justified to expect a higher extent of segregation for wider distributions [9]. On the other hand, newer research is contradictory on this topic, depending on the studied materials and the segregation mechanism. For instance, for model non-pharmaceutical blends in vibrating systems or in heap formation tests, it has been found that mixtures of a broader size distribution may actually be more resistant to segregation [15,16]. This was explained by reduced differences in particle mobility when intermediate-size fractions can be found interacting with small and large fractions. On the contrary, in pharmaceutically relevant studies, the span value of the size distribution either did not correlate with fluidization segregation indices [17], or the ratio of d_90_/d_10_ was positively correlated with the rolling segregation index [14].

Other blend properties may also play a part, although they are considered secondary to size-induced demixing [9]. Differences in ingredients’ particle shape may promote segregation, and high flowability of spherical particles might contribute to this as well. For mixtures with similar particle sizes, a density difference may result in trajectory segregation, as heavy particles for example can sink to the bottom or gain rolling momentum [9]. Another factor which complicates the relationships may be a blend’s tendency to aggregate—in such an instance, the aggregate’s size and durability instead of the primary particles will govern the segregation tendency.

It is interesting to note that a few mathematical models correlating the API particle size distribution to the predicted content uniformity of dosage forms have been introduced. For example, Rohrs et al. proposed a nomograph to determine an acceptable value of the mean diameter (d_50_) for a given dose and content’s standard deviation [18]. Hilden et al. reviewed the existing models and proposed an improved, more complex model, successfully correlating the influence of individual particle size distribution bins on tablet content uniformity, in which RSD can be calculated based on the API’s dose, true density and D [3,6] value describing the particle size distribution regardless of the curve shape [19]. The details are, however, outside the scope of this work, as is the mathematical modeling of physical segregation fundamentals, reviewed for instance by Tunuguntla et al. [20].

### 2.2. Segregation Testing

**Figure 2:**
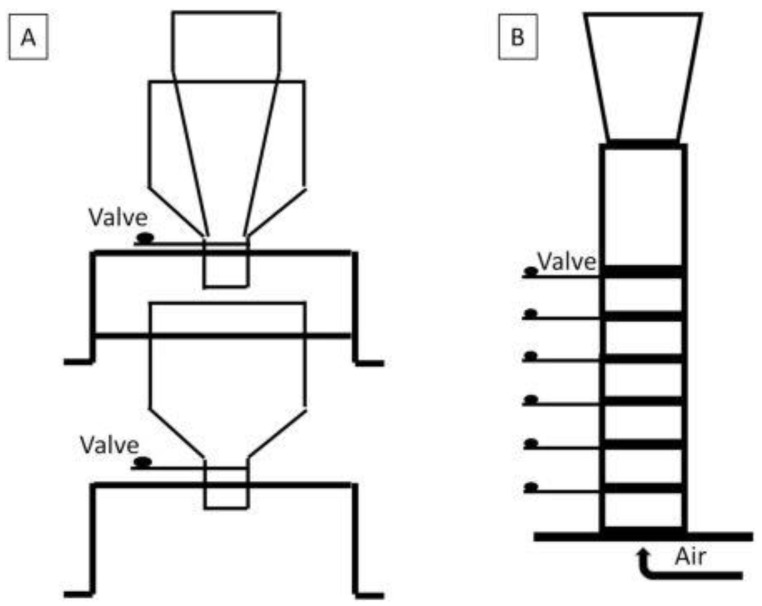
Schematic representation of segregation testers: (**A**) sifting; (**B**) fluidization. The depicted fluidization assembly is a modified version of the ASTM tester with 3 additional compartments. Adapted with permission from [21]; published by Elsevier, 2019.

A blend’s susceptibility to demixing may be evaluated with sifting or fluidization testers according to ASTM standards D6940 and D6941, respectively. Briefly, the former consists of two hoppers; the blend is loaded into the upper one, ensuring mass flow due to its steep angle, and discharged into the lower hopper with funnel flow characteristics, from which samples are collected for analysis upon the release (Figure 2A). On the other hand, the fluidization tester is a compartmentalized tube where the blend is loaded and airflow is introduced (Figure 2B); subsequently, valves between compartments are closed and the composition of the deposit in each section is analyzed. In recent years, several slight modifications of these standard methods [21,22,23,24] or even alternative equipment and protocols for segregation testing in pharmaceutical blends have been described.

For example, one study evaluated the sifting and fluidization potential for blends obtained via various granulation methods, achieving qualitative correlation of the results and tablet CU. For the sieving test, the ASTM assembly was used, whereas the custom fluidization setup consisted of a vertical 6-foot polycarbonate tube (1.5 inch I.D.), fed from a large funnel and emptied into a tightly fitting bottom hopper of the sifting segregation tester. The outcomes were in agreement with the authors’ predictions, as the blends with the highest segregation tendency were the ones with significant differences in granulation and external phase particle size. The segregation could be mitigated via the joint granulation of API and excipients [25]. In one of earlier approaches to alternative segregation testing, Abatzoglou and Simard measured mixing/dispersion tendencies during the flow of binary cohesive and non-cohesive mixtures, as the reverse of the segregation phenomenon. The test was carried out on fully segregated granular mixtures in bins/silos, where the ingredient consisting of finer particles was placed as a ‘pulse injection’ between two layers of coarser particles and their concentration at the bin outlet was measured as a function of time. The easier the penetration of the powder bed, the higher segregation tendency, which is described by the residence time parameter and its variance [26,27].

Other testing approaches have relied on entirely different apparatuses. For instance, Römerová et al. have proposed a simple device consisting of two 25-cm-long pipes (I.D. 2.5 cm). One is placed on top of the other with a closed clutch blocking the flow, loaded with the tested material, which is then emptied into the bottom tube. In the following cycles, both parts are alternated, whereas the samples are collected and the API content, as a function of location in the powder bed column, is determined. The novel tester is capable of detecting both percolation and fluidization, and was proven to discriminate between batches with different segregation potential, related to the API’s particle size and morphology [28]. In another study, the powder rheometer was identified as a potential tool for detecting segregation, as the measured value of flow energy correlated with the segregation index of binary mixture consisting of ingredients with different flow properties [29]. Another example of adjusting a different apparatus for this purpose is the use of an automated powder dispenser, in which a blend is subjected to vibration and stirring in the chamber and samples for analysis are repeatedly dosed into vials. As the authors point out, the advantage of this method is its suitability for high-throughput screening with a significantly lower use of materials than in typical segregation testers [30].

Although the majority of such studies focus mostly on sieving or fluidization, a ‘QPM’ tester has also been developed to assess surface rolling segregation, in which material is discharged from a cubic mixer to a trough inclined at the angle value corresponding to the tested blend’s angle of repose and separated into sections enabling the quantification of the heap’s composition. The tester has served to find positive correlations between rolling segregation index and blend properties calculated by dividing the particle size ratio by the bond number (which in turn is defined as the ratio of a particle’s adhesion force to its gravity). It has also helped to establish that spherical particles have more of a propensity for surface segregation than angular ones [14,31].

## 3. Blend Segregation Phenomena at Different Stages of the Tableting Process

It must be acknowledged that the in-depth direct investigation of particles’ behavior in real industrial equipment and manufacturing processes is challenging, if not impossible [32], although process analytical technology (PAT) tools such as near infrared (NIR) spectroscopy have enabled real-time monitoring of API concentration changes throughout unit operations, making it possible to assess blend or final product content uniformity inline or online and observe potential issues during the process [33,34,35]. Due to this, pharmaceutically relevant studies on exact demixing behavior rely mostly on simulated, simplified experimental setups, or employ computational methods such as discrete element modeling (DEM). It is necessary to note that current capacity limitations require simplifications in DEM simulations, such as scaling up the size of modeled particles when compared to real pharmaceutical blends or challenges with modeling interactions in multicomponent, polydisperse mixtures [36]. Nevertheless, DEM can be considered a valuable tool in understanding the mechanistic details of powder flow, particle trajectories and segregation in different geometrical equipment setups and under stresses during blend handling in tablet manufacturing.

The following sections address studies on demixing during three basic operations or handling events when an initially well-mixed blend may segregate in traditional batch processing—the blend transfer from a container to a tablet press, blend discharge from a hopper or a container, and the blend behavior during die filling in the tablet compression process.

### 3.1. Blend Transfer from Bulk Container to Tablet Press Feeder

The influence of the blend transfer method on segregation and content uniformity in pharmaceutical manufacturing appears to have gained relatively little attention. In conventional batch processing (for continuous manufacturing, see Section 4.3), a powder mixture can be conveyed from an intermediate bulk container (IBC) to a tablet press in three basic ways. The first and most common solution is gravity-driven transfer, where an IBC is located on an upper level over a tablet press and the blend is sent downwards to the press’s feeding hopper through a pipe or a chute. In such cases, sifting or trajectory segregation might take place during the IBC discharge, or particles may be submitted to air entrainment/fluidization during the free fall in the chute. The second method of blend conveying is pneumatic transfer via vacuum suction. Although flow problems or percolation are generally eliminated with this approach, the fluidization of fines in the pneumatic line might still occur and affect the API distribution in the press feeding hopper, although this effect can be mitigated by connecting the conveying line tangentially to the side of the hopper instead of centrally [12]. Finally, a possible method of blend transfer is simple manual scooping, in which an operator loads the hopper with portions taken from IBC. Although this approach is labor-intensive and may potentially suffer from poor repeatability or excessive dust generation, it may reduce the stresses which normally provoke sieving or fluidization in other transfer methods.

To date, fluidization/air entrainment segregation during the gravitational transfer of pharmaceutical powders through chutes has been the subject of a few papers. Liss et al. describe a setup with an open-top vertical pipe, closed with a knife valve and fed with binary mixtures of clearly different particle sizes (dicalcium phosphate 150–425 µm with microcrystalline cellulose, MCC 53–90 µm, as well as mixtures of various size fractions of model glass beads). Segregation (the content of fine and coarse particles) was studied as a function of the height of the fallen sediment. Different pipe diameters (0.5–4 in.) and lengths (6 or 8 ft) were compared and it was found that the topmost layer of the sediment was consistently rich in fines, whereas the bottom layer was fines-depleted, and the higher the ingredients’ size ratio, the larger the extent of segregation. The pattern of vertical segregation suggested that the mechanism responsible was indeed fluidization or particle entrainment in air. Segregation was more pronounced for the longer chute, as a longer free fall increased the time for which drag forces of the air counter-current acted on fine particles. The study also proved the existence of a certain critical minimal pipe diameter for a given blend, which induces flow instability as inelastic collisions between particles and agglomerate formation are promoted. Larger pipe diameter values lessened the extent of segregation [37].

In another study, a problem of lower API content at the end of the direct compression process was identified and attributed to segregation during discharge from a container through a sealed vertical chute. When the powder was gravitationally transferred through the pipe, the air residing in it was displaced upwards, which caused the permeation of the powder bed and the suspension of fine particles. The blend, which contained API particles of larger size in relation to excipients’ particles, was subjected to air elutriation tests according to the ASTM standard, which confirmed the occurrence of air-induced segregation. This led to coarse API particles being discharged into the tablet press sooner and the accumulation of fine excipients reaching the press at the end of the compression run, which explained the changes in tablet potency over the course of tableting. Additionally, the impact of powder flowability and cohesiveness was examined with the use of an annular shear tester and the results indicated that free flowing, less cohesive mixtures are more susceptible to air-related segregation during gravitational transfer through a vertical pipe [38].

On the other hand, Jaklič et al. have pointed out the drawbacks and unrealistic assumptions of the abovementioned studies, such as the lack of an airtight chute bottom, high humidity and no possibility of electrostatic interaction between the chute and flowing material in [37] or the occurrence of different flow regimes in the fast fluidization air elutriation test described in [38]. The authors investigated the particle size distribution of sediments for seven types of single pharmaceutical powders (lactose monohydrate, microcrystalline cellulose MCC102 and MCC200, hypromellose, spray dried lactose, dicalcium phosphate anhydrous and granulated paracetamol) after a drop in a laboratory-scale 120-cm-long vertical glass chute with a diameter of 7 cm and an airtight closed bottom. The results confirmed the predicted accumulation of fine particles in the top layer and the abundance of coarse particles in the bottom layer of the sediment. The extent and exact pattern of segregation depended on material properties, but a universal limit value for large or small particle accumulation at the bottom or top, respectively, was observed, that is 46% of the d_90_ value. The comparison of the obtained results with the experiments carried out with a non-airtight chute bottom confirmed the role of the escaping air counter-flow on the extent of segregation. Velocity gradients for particles of different sizes increase under its influence and local turbulence might occur. The authors also underlined the dependence of the escaping air flow on powder cohesivity and on chute diameter, as wide pipes enable the air to escape through steady channels, limiting the contact time with the powder mass. On the other hand, segregation might be counterbalanced in a way that is difficult to predict due to cluster formation and attractive electrostatic and van der Waals forces. Jaklič et al. concluded that the segregation of small particles is mainly influenced by the presence of larger particles, whereas for medium and large particle segregation, bulk and tapped densities play a part [17].

**Figure 3:**
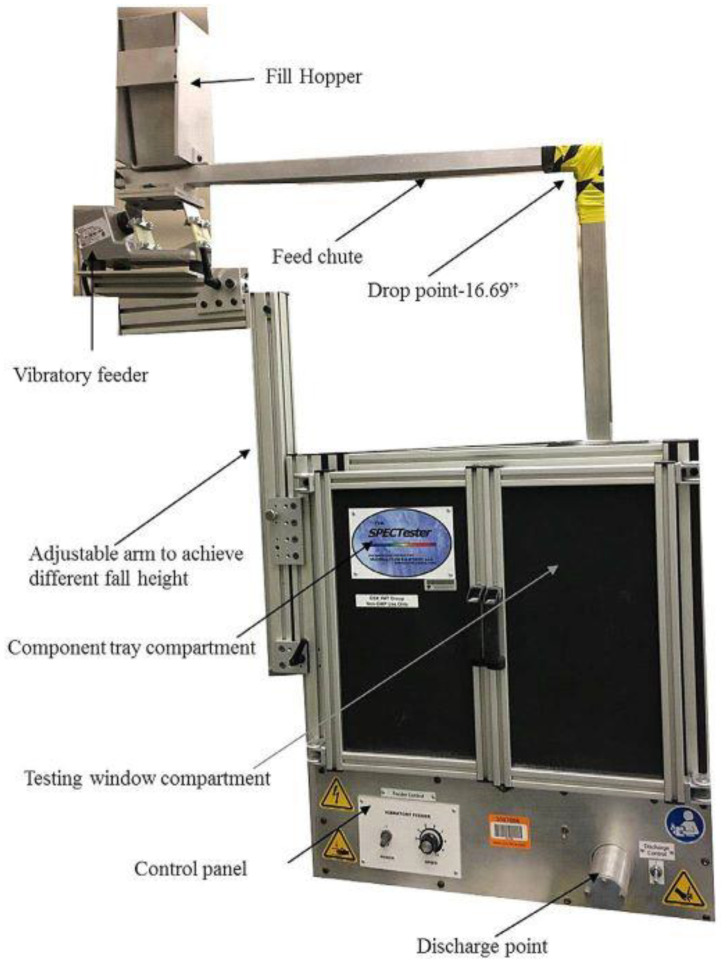
Custom vibratory tester for the simulation of different segregation mechanisms as described by Desai et al., adapted with permission from [39]; published by Elsevier, 2020.

In a recent study, two real formulation blends containing APIs with markedly different particle sizes (one smaller or comparable to diluents, the other markedly larger) were subjected to ASTM fluidization tests and to a vibratory segregation test in a custom setup (Figure 3). In the latter method, the blend flows out of a vibratory feed hopper through an inclined feed chute and falls through a 16.68-inch-long drop chute to form a heap, where the compound distribution is analyzed using an NIR probe to assess the tendency towards segregation. This complex assembly is capable of inducing the segregation of different mechanisms, such as sieving in the hopper, trajectory segregation due to different adhesion and flow on the inclined chute, and air entrainment in the drop pipe. The experiments confirmed that an API’s axial and radial segregation profile was a combination of different mechanisms, with the finest active ingredient being prone to both sifting and fluidization, whereas the blend with a larger API:excipients size ratio was bottom-enriched due to rolling. The authors pointed out, however, that such a test cannot be considered a simple prediction of a blend’s behavior in the real manufacturing process, as the presence or absence of vibration affects the propensity for demixing. Interestingly, the large API was found to be susceptible to considerable segregation during fluidization tests, which correlated with practical content uniformity problems during manufacturing involving the free fall of the blend [39].

As the researchers who describe powder segregation in vertical chutes have pointed out, the extent of air-induced gravitational segregation can be reduced through optimum equipment design. Avoiding long drop heights limits the time for the effect of differential drag forces to take place, and increasing pipe diameter might be beneficial. Furthermore, minimizing the discharge rate by decreasing the container outlet diameter or using a rotary valve to drop the powder in small increments reduces the extent or rate of air displacement from the chute. However, the most important and widely employed method is the creation of an alternative way for air to escape than through the powder mass, which can be achieved by installing an external venting system. Additionally to equipment modifications, increasing powder cohesivity and reducing its flowability could be considered where possible [12,17,37,38,40]. However, when such measures are not feasible, simplistic solutions such as changing the transfer method to manual loading may be applied to mitigate segregation. In an industrial case study on a direct compression blend containing two APIs, two conveyance approaches were compared—direct powder scooping from an IBC with a stainless steel bowl for manual loading into a press feeder, and discharge from an IBC into a drum with subsequent scooping, imitating the typical gravitational transfer. Although the tablets compressed from blends delivered in both ways complied with Ph. Eur. requirements, one of the APIs exhibited a significant increase in content towards the end of the process and higher AV and RSD values when fed after a simulated gravitational transfer. On the other hand, the issue was not observed when manual loading was applied, although the exact demixing mechanism was not investigated [41].

A DEM simulation study which can be considered broadly related to blend transfer was performed by Zhang et al., who investigated the particle mixing index and distribution of particles according to their velocities and trajectories in a binary mixture during filling into a conical hopper. The process could be divided into three stages: the initial filling of a well-mixed region in the bottom part, pile-up, and steady filling, in which segregation took place. During the last phase, large particles were found to roll in the heap at larger distances towards the hopper walls, whereas smaller ones remained in the center. The extent of segregation (as per the particle mixing index) was more pronounced in the higher hopper regions, as well as close to the walls. In general, particle trajectories were governed by bouncing in the case of the small fraction, and rolling for the large one, whereas the mixing index was dependent on the assumed sliding friction and rolling friction coefficients [42].

Regarding the topic of material transfer in solid dosage form manufacturing, it might also be noted that segregation events may potentially be induced by changes in the original particle size due to attrition. For instance, a developmental API has been found to undergo bulk fragmentation and a size reduction during the feeding in a horizontal screw feeder even though it resisted comminution in a tumbling blender and conical mill [43,44] Other reports have described the granules’ attrition in continuous twin screw granulation lines, namely during the transfer of wet granules to the fluid bed dryer and at the transfer line of dried granules. The effect of a diminished ‘oversized’ fraction and an increased fine content was more pronounced for pneumatic conveyance lines than for gravitational transfer, and the susceptibility of the dry material to breakage was related to formulation composition and final moisture content, so that the drying process needed to be optimized for the generation of sufficiently solid bonds for the material to resist comminution [45,46]. However, the aforementioned papers did not assess any segregation events relating to the attrition.

### 3.2. Blend Segregation in Hoppers

Ketterhagen et al. applied DEM simulations to investigate the flow and sifting segregation in discharge hoppers of various geometries—cylindrical, wedge-shaped, concentric and eccentric conical [47,48,49]. The first study of the cycle yielded a good prediction of the experimental segregation of bidisperse glass spheres. Apart from hopper angle (15°, 55° and 90°) and shape (cylindrical vs. wedge-shaped with rectangular cross-section), the authors also investigated the simulated influence of fines content, particle size ratio and the method of hopper filling. Among them, the dual hopper filling method, as in the ASTM D6940 test, was considered the most significant in the industry and resulted in a different discharge profile when compared to the loading of a well-mixed blend. When the lower hopper was filling, fine particles dispersed due to collisions with the material bed surface and concentrated at the hopper walls, which is the reason for non-uniform flow during discharge and the fine-depleted central region exiting first from the bottom hopper. On the other hand, in the case of well-mixed loading, a cylindrical hopper induced greater segregation, and the higher the fines content, the lesser the extent of demixing due to there being fewer void spaces to percolate through. The flow patterns and segregation tendency were influenced by the interplay between the wall angle and the particle size ratio, with different angles proving to be the most beneficial at different combinations of size ratios and fine fractions [47]. The second of the studies conducted by Ketterhagen et al. on a simulated quasi-3D wedge-shaped hopper confirmed the importance of the particle size ratio and fine fraction for the segregation potential, and a limiting fine fraction value was found to be the cutoff boundary crucial for the occurrence of segregation in the studied conditions. The aforementioned value depended on the particle size ratio of components (for instance, for a ratio of 4.3, the limit was calculated as 48.2%). Particle–wall friction also played a part, as high values caused the fine particles to accumulate at the walls and discharge only at the end of the process. Moreover, recommendations were confirmed for hopper design aimed at ensuring mass flow and uniform discharge (steep walls, a large aspect ratio, a wide outlet, using low-friction inserts or reducing wall friction by polishing/electroplating) [48].

**Figure 4:**
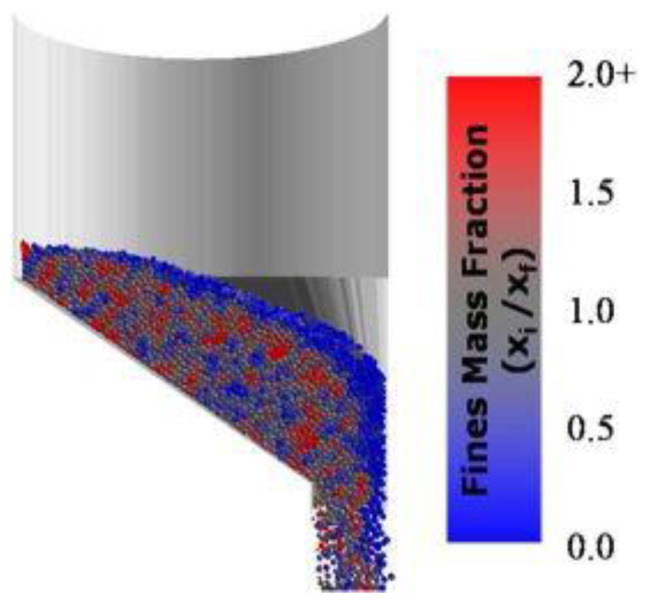
Segregation during discharge in an eccentric hopper, with the red color denoting the fine-rich fraction, and blue denoting the coarse fraction, according to discrete element modeling (DEM) by Ketterhagen et al., adapted with permission from [49]; published by Elsevier, 2010.

Although the aforementioned papers seem applicable to many engineering fields employing bin discharge, the final study of this series is potentially most relevant for the pharmaceutical industry, comparing the effects of concentric vs. eccentric hoppers, which is a common design for tablet presses, by modeling single and two-component systems. The results indicated no difference in the segregation extent, although the eccentric conical hopper was characterized by an up to 35% higher discharge rate. However, eccentric hoppers pose the risk of an asymmetric horizontal segregation profile, as fine-rich material is discharged close to the inclined wall, whereas fine-depleted material moves down the vertical wall (Figure 4). The authors point out the potential problem of non-uniformity in cases in which the powder is subsequently split after discharge, e.g., in a double-sided rotary tablet press or in a press feed frame in which paddles rotate in opposing directions. A comparison of different simulated feed hopper designs revealed that the segregation index may be reduced via the choice of a concentric geometry, and—for eccentric hoppers—a steeper wall angle or lower wall friction [49].

In a more recent study, Zhang et al. used DEM to simulate percolation during discharge from a conical hopper of different angles (30°–180°) for mixtures of higher size ratios than are usually explored (4–14.3). The emptying process could be divided into three stages: initial, in which fines dominated; transitory; and final, in which mostly large particles flowed out. It was found that at higher size ratios, segregation was more pronounced and the time required for the complete discharge of small particles accumulated at the bottom was shorter. With unchanged volume fractions of different sizes, this is explained by the considerable void volume available at high size ratios between the network of large particles, where fines experience fewer collisions and are essentially subjected to free fall conditions at high velocities. On the other hand, at lower size ratios their mobility is restricted; therefore, it was found that for ratios below the value of six, velocity gradients were reduced and sifting segregation was less significant [50].

**Figure 5:**
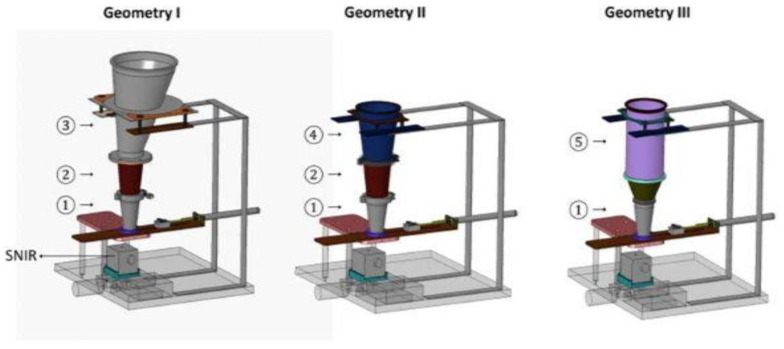
Possible hopper geometries: I—asymmetrical conical; II—symmetrical conical; III—cylindro-conical, as studied by Abatzoglou et al., adapted with permission from [52]; published by Elsevier, 2014.

Another group of studies has focused on the NIR monitoring of radial and axial distributions of particles flowing in hoppers. Castellanos Gil et al. determined the residence time distribution of lactose particles placed as a pulse injection between layers of microcrystalline cellulose in a Quick-clamp adapter tube and found that the RSD of this parameter (and consequently segregation) increased with an increase in the particle size ratio [51]. Abatzoglou et al. compared the flow and velocity of fully segregated (by pulse injection) mixtures of lactose and MCC in pilot-scale hoppers of different geometries (Figure 5), as well as investigated the influence of the powder bed mass and height. The results indicated that particles’ residence time and shear conditions are dependent on their position in a hopper, and powder radial and axial velocity profiles are strongly influenced by hopper shape. A conical hopper favors mass flow as opposed to a symmetrical cylindro-conical hopper, whereas an asymmetrical conical hopper may force the radial segregation of particles along its vertical and inclined wall [52].

Other studies applied NIR for inline real-time monitoring of blend and content uniformity during the tableting process, in which variations in CU were attributed to segregation events at the level of the feeding hopper. In a study by Karande et al., direct compression tablets of chlorpheniramine maleate passed the USP content uniformity test when sampled in a stratified manner, although inline NIR monitoring detected an increase in tablet potency after 30 min of tableting. The authors interpreted this as a result of segregation due to differences in particle size and the density of components, as well as the hopper geometry. They concluded that a bin hopper with a tapered end initially ensured mass flow, but channeling in the core region occurred later. Rolling segregation took place, in which denser particles (lactose coated with API) moved downwards faster than lighter MCC particles [53]. Similarly, in another study an NIR probe mounted in a tablet press feed frame detected an increase in the model API signal at the end of the process, which correlated with an increased but still acceptable API content in tablets. Wahl et al. attributed this to particle size segregation in the feeding hopper (large API crystals flowing out last), exacerbated by tablet press vibrations and fine powder attrition due to the force of the feed frame paddles [54].

**Figure 6:**
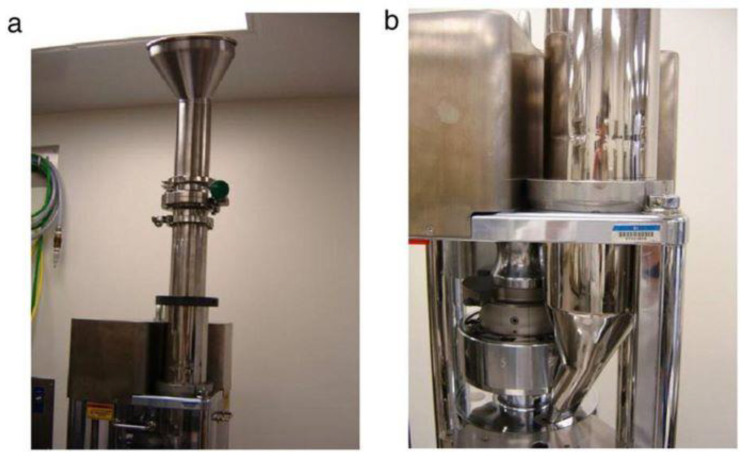
Extended-feeding hopper setup as designed by He et al.: (**a**) overview; (**b**) close up. Adapted with permission from [23]; published by Elsevier, 2013.

He et al. correlated the results of aspirin CU in directly compressed tablets with the results of blend segregation tests conducted in ASTM sifting and fluidization testers. For the tableting experiments on laboratory scale, the authors used a specifically designed extended hopper to force segregation due to both mechanisms (Figure 6). The intention was to imitate the risk conditions of an industrial scale: fast bin filling; a long free fall height and a sharp outlet angle, promoting funnel flow. The effects of powder particle size and cohesion were examined by varying the API milling method and by coating aspirin particles with Cab-O-Sil M5P. The obtained results contradicted the simplified view of particle size difference being the deciding factor for segregation. The blend with the least difference in the size of aspirin and excipients (lactose and MCC) displayed the highest segregation tendency. The significant increase in tablet potency near the end of the tableting process would cause the tablets to fail CU criteria (RSD > 6%). On the other hand, blends with smaller, jet-milled API particles showed lower segregation tendencies and better CU. The authors concluded that for segregation to occur, particle interactions are an important factor in addition to particle size differences. The MCC used in the blends was characterized by a porous surface texture and acted as a carrier for API particles. Adherence to MCC was facilitated in the case of smaller particles, which prevented the segregation of aspirin from other components. Powder cohesion, however, did not appear to influence the segregation tendency [23].

### 3.3. Blend Segregation during Die Filling

Several studies have been carried out on the powder flow during die filling and can be divided in two groups. First, those with an experimental or computational setup of a stationary die and moving feeding shoe, which corresponds more or less exactly to the compression process with a single-station eccentric tablet press. Second, studies with a stationary feeder and moving die, which is more relevant to the industrial setting of tablet manufacturing on a rotary tablet press. Among the latter group, quite a lot of consideration has been given to feed frame design and operating parameters.

#### 3.3.1. Studies on Segregation in Stationary Die and Moving Shoe

Many of the studies employing a horizontally moving shoe discharging the blend into a stationary die do not concern segregation and content uniformity, but focus on the flow pattern and mass uniformity. This type of research has relied on the notion of critical shoe velocity (maximum velocitywhich results in complete filling of the die cavity) and often has compared the mechanisms of gravity-driven fill and suction fill. The results indicated a considerable influence of the air residing in the die on the powder flow and the extent of filling, as better flow and complete mass deposition in the die was easily achieved under vacuum conditions [55,56,57,58]. Specific flow patterns and segregation phenomena in this type of setup have been the focus of a series of papers by Guo et al. [59,60,61], who used the model moving shoe and stationary die setup in the 2D simulations with DEM and computational fluid dynamics to investigate the segregation of a binary mixture with ingredients of similar particle sizes and different densities (2100 vs. 7800 kg/m^3^). In the first study, the filling of three rectangular dies with differing dimensions (termed ‘narrow’, ‘square’ or ‘deep’) was compared with the assumption of either a stationary or a moving shoe. When the shoe was immobile, the horizontal distribution of particles in the die was the same as in the filling shoe, whereas vertical segregation depended on the die design. The deeper the die was, the higher the concentration of lighter particles at the top of the deposit, as their rebounding from the bed was characterized by higher momentum. The presence of air exacerbated the effect, with the counter-current delaying the fall of light particles, and additionally provoked segregation in the ‘narrow’ and ‘square’ geometry. For the moving shoe setup, both vertical and horizontal segregation took place. Particles were characterized by different inertia and air sensitivities, and interparticle collisions caused them to gain different velocities, so that the lighter particles rebounded and enriched the powder stream at the front. In general, contrary to the stationary shoe setup, powder beds filled into dies regardless of their shape were enriched with light particles at the bottom due to them sliding more readily because of lower inertia; the presence of air increased the vertical segregation. In the case of horizontal demixing, the distribution depended on the die type and flow pattern and the highest segregation index was found for the square die, which related to its highest volume [59]. A follow-up study found that particle segregation of binary mixtures of similar size increased with their density ratio. The extent of horizontal segregation could be reduced at higher values of shoe velocity, as the flow pattern changed from ‘nose flow’ to more favorable ‘bulk flow’ [60]. The same setup was extended to 3D DEM (resulting in a cubic die model) to investigate the effect of a binary mixture with different particle sizes. In the case of a moving shoe, no segregation difference was found with the simulations in a vacuum and in the presence of air, as the residual air was able to escape before complete die filling. Powder shearing during shoe movement affected the discharge and promoted the percolation of fines. A combination of sifting and rolling along the free surface during the filling resulted in the accumulation of fines at the bottom and near side of the die, whereas larger particles prevailed at the far end [61].

An experimental investigation on feeding a pharmaceutically relevant blend (aspirin 350–425 µm and mannitol 107–125 µm) from a moving shoe into a stationary cubic die under gravity and suction was carried out by Zakhvatayeva et al. Apart from the presence or absence of air, different drug loads (10–45%) were studied, and API concentration as a function of fill depth and distal or proximal location was measured to calculate the segregation index. Additionally, the authors intended to compare shoe velocities; however, the conditions of high speed cannot be considered relevant to real processes, as one shoe passing was insufficient for complete die filling and a sequence of three nose-bulk flow filling cycles was forced; therefore, only low-speed results are considered here. At high API contents, practically no vertical or horizontal segregation was observed. On the other hand, at 10% drug load under gravity filling a pronounced increase of aspirin content with fill depth was observed, which was explained by the fluidization of finer, air-sensitive mannitol concentrating at the top. Accordingly, suction fill conditions eliminated this vertical segregation. On the other hand, the horizontal distribution of ingredients was uneven regardless of the presence of air, but no clear trend was found [62].

An interesting example of particle segregation in a stationary die-moving shoe setup is an experimentally validated DEM study on different feeding shoe geometries, which varied by width, toe height and bridge angle. Fine fractions ranging from 25% to 75% were investigated in several prototype designs and, regardless of geometry, the 50% blend displayed the highest segregation tendency. The extent of demixing for the same mixture was dependent on the shoe configuration, but a common pattern was generally observed, i.e., fines accumulated at the location of their fall, whereas coarse particles slid further. Segregation was observed in the shoe itself upon filling even before the movement sequence started. Nevertheless, the study demonstrated that the shoe design configuration can be optimized with the help of DEM modeling, and proper inserts, the aim of which is to reduce friction at the shoe bottom or to increase the confinement and immobilize the material at the top section, are able to cut the segregation index even by up to 50% [63].

The DEM simulations of an eccentric tablet press setting have also been extended to minitableting dies filled gravitationally from a moving shoe (45 mm/s) with a polydisperse mixture. Since particle mobility in the die was restricted by the minitablet dimensions, it was found that the segregation in the filled material bed was a direct product of the segregation pattern in the shoe. Therefore, both vertical percolation and horizontal segregation were observed. Larger particles rolled faster over the free surface in the shoe, deposited at its front and entered the die first, resulting in the large-enriched bottom of the minitablet. Interestingly, the more distant the die was from the initial shoe position, the less pronounced was the difference between the top and bottom. The study also showed that at higher friction, segregation was more likely, and demixing could be mitigated by increasing the loaded mass into the shoe, as denser particle packing restricted percolation [64].

#### 3.3.2. Studies on Segregation in Rotary Tablet Presses

##### Gravity Feeding

**Figure 7:**
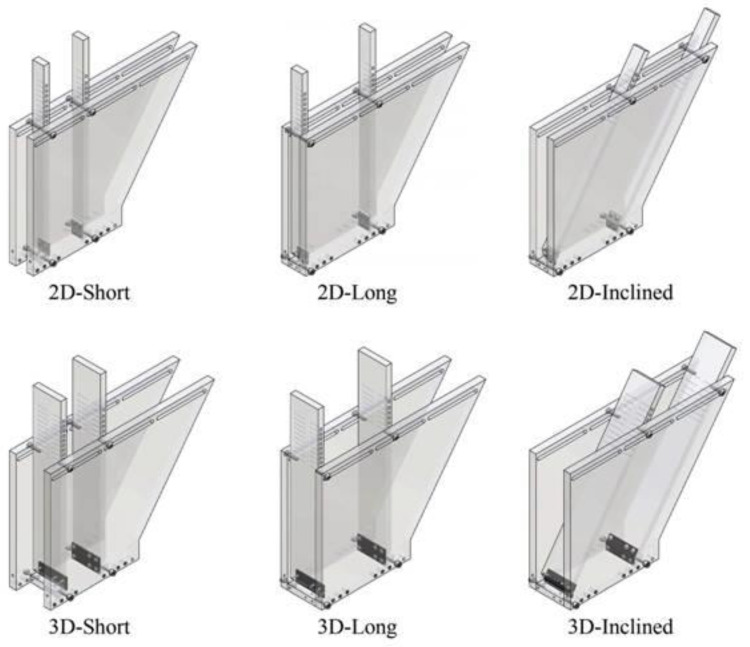
Feeding tank configurations as compared by Ramírez-Aragón et al., adapted with permission from [65]; published by Elsevier, 2018.

The blend for compression in rotary tablet presses can be delivered from a feed hopper to moving dies on a rotating table either by means of a gravity feeder or a force feeder (feed frame) with rotating paddles. Segregation in the former kind of setup has been investigated by a couple of research groups. For instance, Ramírez-Aragón et al. applied experimentally validated DEM to compare geometries of a gravitational feeding tank with differing lengths, widths and wall slopes (termed ‘short’, ‘long’ and ‘inclined’ designs, Figure 7). The ‘inclined’ configuration was found to induce the highest extent of segregation, especially in the wider variant. Although the sieving segregation index was similar between the ‘short’ and ‘long’ designs and the fines content in the filled dies was stable throughout the process, the ‘inclined’ configuration induced rolling segregation. As a result, the coarse particles slid to the front, whereas fines accumulated near the inclined wall and emptied first into rotating dies, which translated to an excess of fines in the first tablets and a decreasing trend with process time. In general, a comparison of tank geometries and different filling methods demonstrated that segregation can be reduced to the highest extent by continuous feeding of a ‘short’ tank, which ensures mass flow [65].

Another application of DEM for the simulation of gravity feeding in a rotary press is the study of particle discharge into dies from a rectangular box filled from a feeding pipe via an inclined chute. The flow and turnover of large and fine fractions was studied throughout different box sections, and the particle size distribution inside the box fluctuated throughout the tableting process due to sifting and competition between die filling and mass refilling from the feeding pipe. However, the most interesting aspect of this paper from the perspective of the current review is the simulation relating the behavior of a blend containing 8.5% API and MCC to the content uniformity of the filled dies. It is noteworthy that these results diverged from more general simulations of fine vs. coarse particles in the same study, reinforcing the importance of considering the whole blend’s size ranges in relation to flow and segregation phenomena. As the modeled compression progressed, the API—as the smallest fraction—accumulated within the box and as a consequence the drug content in the tablets decreased with time, causing the failure of the acceptance value (AV) calculation. Passable CU results and lack of trends were obtained only when a lower particle-wall friction coefficient was simulated, although the authors were cautious in extrapolating this finding beyond the setup used [66].

A setting that is probably more relevant for real manufacturing in rotary presses has been presented in another study, in which suction due to lower punch movement was considered and different die speeds (850–2000 mm/s) were compared for sucrose granules via DEM and its experimental validation. The die was divided vertically and horizontally into several sections for the assessment of segregation indices and it was found that segregation was the most extensive at the frontal and lower parts of the die. Not surprisingly, the segregation increased with the decrease in the particle size of the fine fraction. It is interesting to note that according to the response surface map in this particular study with gravity feeding, the die table speed did not influence the segregation index [67].

##### Force Feeding

Of more relevance to real tablet manufacturing via direct compression in rotary tablet presses are studies on the powder behavior in feeding frames, which have also been reviewed by Sierra-Vega et al. [68]. Therefore, in this work only the significance of the feed frame design and operating parameters for segregation and content uniformity are highlighted.

**Figure 8:**
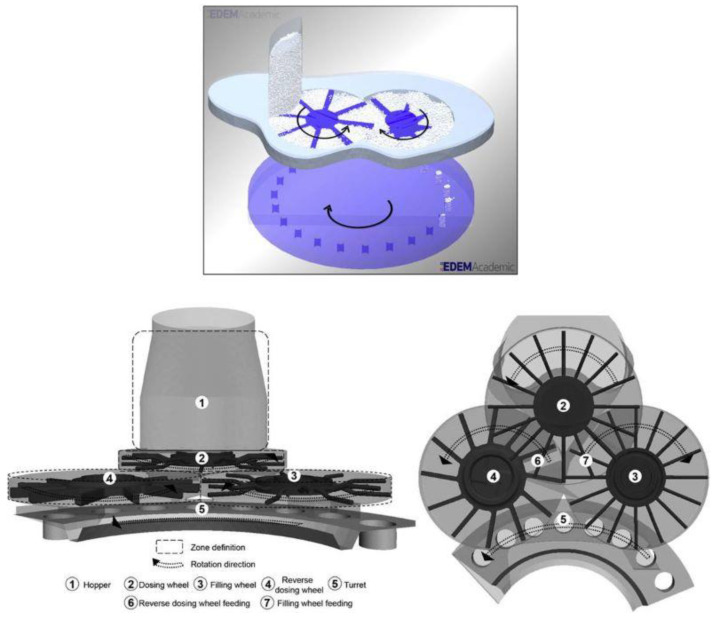
(**Top**): Schematics of a two-paddle feed frame. Adapted with permission from [69]; published by Elsevier, 2014. (**Bottom**): Schematics of three-paddle Fill-O-Matic feed frame. Adapted with permission from [71]; published by WILEY-VCH Verlag GmbH & Co. KGaA, 2018.

Mateo-Ortiz et al. used DEM to simulate the phenomena in the two-wheel feed frame (Figure 8, top) and validated the results experimentally on a mixture of API granulation, lactose and magnesium stearate. The study investigated the effect of paddle and die disc table speed (24–72 and 29–57 rpm, respectively), as well as estimating the role of each of the three frame exits on the filling mass. Among other findings, the authors concluded that sifting segregation might occur in the feed frame depending on the location, with its extent increasing with a slower paddle rotation, which gives time for particles to rearrange according to their size and potentially impacts the segregation in dies, where small particles cumulate at the bottom [69]. The same equipment and operating speeds were used in another study by the same group, in which residence time distribution was DEM-simulated and validated experimentally for a pulse injection of naproxen (median size: 28 µm) into a lactose blend (median size: 125 µm). A faster paddle speed shortened the residence time, whereas faster table speed delayed the particle exit by changing the dominant discharge location to a different frame opening. In general, small particles were found to empty faster into dies, whereas larger ones circulated within the feed frame for a longer time, which was tentatively attributed to oscillatory segregation [70].

Mendez et al. compared the shearing fragmentation and particle size distribution change between a two-paddle feed frame (Manesty press type) and a three-paddle wheel frame (Fette Fill-O-Matic) at different paddle and die disk speeds. The latter consisted of a dosing wheel in the upper section, and reverse-dosing and filling wheels in the lower frame section, with paddles of different geometries (Figure 8, bottom). Based on the example of lactose and roller-compacted placebo blends, it was found that particle attrition and size distribution shifts were affected by the residence time, depending on the frame type, operating speed and die diameter. In general, smaller dies, faster paddle speed and larger volume of the three-chamber feed frame increased particle fragmentation due to prolonged exposure to shearing stress [72].

In-depth investigation into segregation related to Fill-O-Matic performance has been described in a series of papers by Hildebrandt et al. [32,36,71]. DEM simulation was employed for a direct compression blend consisting of six size fractions of excipients and twice-as-small API particles. Although the mixture retained homogeneity in the hopper, sieving segregation took place in the frame, starting from the dosing wheel section (see Figure 8, bottom). Fine API particles percolated to the bottom and were subsequently fed to the next section of the reverse-dosing wheel, where they accumulated. As a result, the blend entering the filling wheel section in the next step, from which material was transferred into dies, was depleted of fines. This was demonstrated by significant deviations from the theoretical API content of 5% in each section. Although the hopper was close to theoretical value (4.7%), the amount in the dosing wheel was already affected by sifting (3.4%), and the accumulation and depletion of fines in the reverse-dosing and filling sections were clearly visible, with values of 10.7% and 2%, respectively. Regarding the API contents in the simulated tablets, due to scarcity in the filling wheel the mean value was significantly lower than the target (4.8% ± 0.8%), but, surprisingly, no trend throughout the process was observed. Potency fluctuations in tablets were considered rather minor in spite of the blend segregation and deviations in the feed frame. This was explained by the mutual compensation of counteracting trends, where the content increase in the filling wheel was simultaneous with the decrease in the reverse-dosing wheel and vice versa. It is therefore interesting to note that the occurrence of segregation even at the last stage of the blend’s fate during the compression process does not necessarily translate into grave practical problems with the content uniformity of tablets [71]. Nevertheless, in a follow-up paper it was verified that the studied polydisperse system failed in terms of CU, with an unacceptably high AV result, which the authors attributed to the large particle sizes relative to die dimensions in the DEM simulations [36].

Particle fate and segregation indices were then explored in depth for each section of the feed frame during the steady state of high speed tableting (turret speed: 40 rpm, paddle: 30 rpm). According to this, the hopper filling was not uniform across the width in the section connected with the dosing wheel, with the right zone experiencing a higher mass flow and API content, although the particle size distribution was close to the theoretical numbers. Demixing occurred further on and led to segregation in the dies, as large particles entered first, followed by small particles, which were exposed to more shearing at the top of the fill. At high rotation speeds, sifting from the dosing to the reverse-dosing zone was reduced; therefore, fines were transferred along with their distribution fluctuations to the filling-wheel zone. Additionally, trajectory segregation was detected in the reverse-dosing section, where coarse particles collected at the outer side and the top free surface. As a result of these complex phenomena, the segregation index of the API calculated for the whole feed frame was high. In the filled dies, the segregation of fines was mainly affected by the size distribution in the reverse-dosing wheel, whereas in the case of coarse particles it was affected by that in the filling zone [36].

The final study by this group compared the previously used cylindrical shape of paddles to the use of a cuboid shape at different combinations of turret and wheel speeds (40 + 60 rpm or 75 + 30 rpm). None of the tested setups resulted in acceptable content uniformity according to AV calculations; moreover, the mean API content in the tablets was 25–39% higher when cuboid paddles were applied. This was an effect of the exacerbated trajectory segregation with this design, as high shearing caused coarse particles to be lifted by the blades and to circulate in interblade zones, accumulating at the hopper exit, especially at faster wheel speeds. Meanwhile, the fine particles of the API were not subjected to circulatory flow and were free to enter the dosing wheel, following the route to the dies. As a consequence, cuboid paddles fed the dies with a higher API dose. In summary, the least problematic setup was identified as a combination of a low turret and a high wheel speed with typical cylindrical paddles [32].

The three-chamber Fill-O-Matic has also been investigated in an experimental study approaching the issue of segregation from the other side. Namely, the use of the feed frame as a mixing unit was evaluated by observing the residence time and content uniformity of a lubricated lactose–MCC blend containing 10% theophylline as a tracer, fed as a well-mixed load or as separate ingredients, at different paddle (30–90 rpm) and tableting speeds (12,500—50,000 tablets/h). Interestingly, in this case, even with split feeding it was possible to achieve the target value of API content and an acceptable CU at 25,000 tablets/h. At lower turret speeds, the results were out of specification, likely because of segregation related to longer residence time in the feed frame. Alternatively, speeding the compression up to 50,000 tablets/h also worsened the CU, which was explained by a poorer mixing capacity at a narrower residence time distribution [73]. The residence time distribution in the Fill-O-Matic has also been studied experimentally for a traced blend of microcellulose, containing three size fractions (below 50, 125–180 and 315–400 µm). Despite similar time profiles for each size, segregation in filled dies was detected, with fines accumulating at the tablet bottom, the largest fraction at the top and the intermediate fraction distributed homogenously [74].

**Figure 9:**
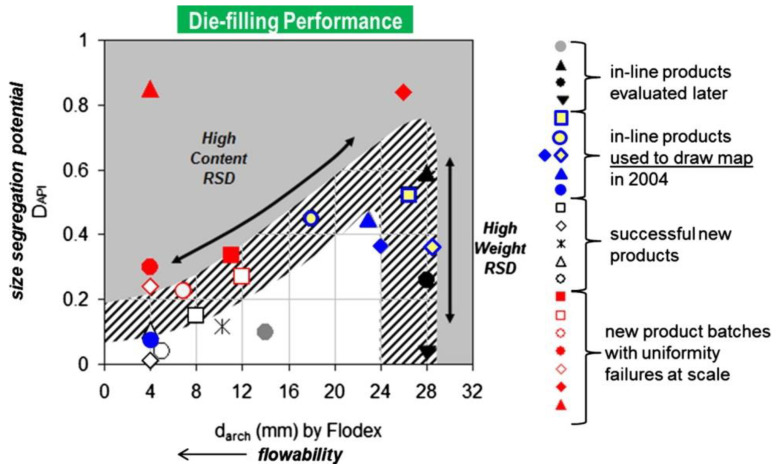
Practical map for estimating the risk of a loss of content uniformity based on a segregation index related to the size and active ingredient fraction in the blend (D_API_) and the critical arching diameter (d_arch_) determined using the Flodex flow apparatus. Gray area denotes high risk (relative standard deviation >5%), white area—low risk, hatched region—marginal risk. Adapted with permission from [40]; published by Elsevier, 2015.

It is worth mentioning a practical industrial study originating from Merck & Co. in 2004, in which a model was developed for predicting the risk of tablet content uniformity loss due to segregation at the feed frame level in a worst-case scenario. Gentzler et al. considered it as the main segregation site, owing to the optimized equipment design upstream of the tablet press feeder, i.e., the mass flow discharge bin and a rotary valve which enabled it to drop powder in small increments through the Y-chute to a double-sided rotary tablet press. Upon researching various direct compression blends, based on measurement of the blend cohesivity and size segregation potential (via sieve analysis and the calculation of a custom segregation index), an empirical map for the estimation of the segregation risk was proposed. The rationale behind this assumes that for typical pharmaceutical blends, polydispersity is the main demixing trigger and most fines are cohesive in nature. According to this, a high tablet content RSD concerns powders with a high drug load and a low critical arching diameter as determined in cohesivity tests, confirming the susceptibility of well-flowing powders to segregation (Figure 9) [40].

## 4. Content Uniformity Improvement and Segregation Prevention by (Pre)Formulation and Processing Choices

### 4.1. Examples of Blends for Direct Compression

Apart from research on segregation during the hopper discharge and die filling of a tablet press, in recent years several development studies have been carried out on the optimization of formulation and processing parameters in order to improve tablet content uniformity by reducing potential demixing. Simple formulation studies confirm a higher segregation tendency with increasing size ratios between excipients and API, as well as at low fine API drug loads [30]. Different crystal habits of the same API have also been compared. For example, fine needle-shaped and granular rod-shaped active ingredients displayed different trends in terms of their content in tablets, even though they were divergent from their respective blend uniformity trends, although the CU was acceptable in both cases [75]. In another study, diverse particle morphologies and the proportion of a given shape of crystals within a particular API batch were found not only to result in different segregation potential, but also to undergo contradictory segregation mechanisms, depending on the applied mixing approach (simple or multi-step mixing with additional sieving) [28].

Muselik et al. examined the influence of calcium hydrogen phosphate particle size and the time of lubricant addition during blending on the content uniformity of directly compressed warfarin tablets. They discovered the significance of both factors, though when magnesium stearate was added in the course of the ongoing blending process, there was no difference in CU between tablets manufactured with fillers of different particle sizes [76]. Yousaf et al. developed generic entecavir tablets of improved CU compared with the original formulation. The effects of API particle size, colloidal silicon dioxide content, preblending method and sieving were examined. An enhanced AV was found for micronized entecavir—double sieving and smaller pore diameter, as well as increased amount of glidant [77]. Xie et al. used the design of experiments approach and principal component analysis to correlate a blend’s segregation tendency to parameters such as aspirin grade, MCC grade and magnesium stearate amount, as well as different physical properties of the blend. The API grade had the strongest statistically significant effect on the concentration ratio of aspirin between the last and first sample discharged during a sifting segregation test, whereas the MCC grade did not affect segregation. These findings confirmed particle size difference to be the driving mechanism, whereas blend flow properties were found to be insignificant in this case [78]. In contrast, a newer study found that the presence of more than 10% MCC in a direct compression blend can be beneficial for the reduction of acetaminophen content RSD in tablets at a drug load of 2%. Nakamura et al. attributed this to the ability to increase interparticular friction and inhibit segregation, based on a discovered correlation between CU and the value of the angle of internal friction for a particular blend. The authors suggested the possibility of using this parameter as a predictor for homogeneity control [79].

### 4.2. Examples of Granulated Blends

Some studies have used the design of experiments approach to compare the impact on model active CU of two tablet manufacturing methods—direct compression and dry granulation-roller compaction. Morris et al. investigated the influence of three factors—formulation (lactose and MCC mixture vs. dibasic calcium phosphate and MCC mixture), drug load and manufacturing method. All batches fulfilled the acceptance criteria, with the lowest variability shown for the roller-compacted lactose formulation, with lactose being a more favorable excipient. Generally, dry granulated batches were characterized by lower content RSD; however, the difference did not reach statistical significance. Similar tendencies were observed in segregation tests of the blends using the ASTM type tester, in which the direct compression mixture segregated only under forced conditions of dual hopper filling [80]. Kushner et al. carried out manufacturability assessments, aimed at establishing the design space for various tablet CQAs, including content uniformity. The examined factors were API type (ibuprofen vs. theophylline), API particle size, drug loading, size ratio of filler and lubricant particles and finally the processing method (direct compression vs. roller compaction). Insufficient tablet CU resulted from direct compression of blends of low drug loading (below 2.5%) and high API particle sizes with more lubricated, coarse fillers. The general conclusion indicated that dry granulation could improve content uniformity, but considering overall tablet CQAs a clear, deciding benefit of roller compaction was not found [81]. Am Ende et al. optimized the parameters of roller compaction and milling—roll force, gap width, sieve size and granulator speed—in order to improve low-dose tablet content uniformity. The excipients in the study were chosen so as to force small-size API segregation on purpose. Pre-optimization, the granulation was characterized by high sifting and fluidization segregation potential (as determined by ASTM testers) and excessive potency in fine granules. The improved distribution of the API content across the granulation particle size distribution also improved tablet CU, which was due to a smaller sieve size, higher roll force and larger gap width [82]. Roller compaction was also proven to be efficient in improving blend uniformity by dispersing API agglomerates [83].

Some studies have also focused on segregation during wet granulation. NIR chemical imaging of blends and tablets was employed to trace the segregation of ethenzamide and lactose upon optimal high-shear wet granulation (HSWG) and overgranulation (as governed by time and impeller speed). Although the optimal process delivered a homogenous distribution of ingredients in tablets, lactose agglomeration was observed for overgranulation during the consolidation phase, in which hydrophobic API separated. Nevertheless, both blend and tablet content uniformity were acceptable [84]. Oka et al. investigated inhomogeneous distributions of ingredients between size fractions after HSWG, in which large granules were subpotent, whereas finer granules contained an excess of acetaminophen. The demixing potential was lower at a higher drug load of 7% (vs. 3%) and a higher impeller speed (300 vs. 225 rpm) [85]. Further exploration revealed that the segregation was initiated at the dry mixing stage before binder addition, regardless of mixing time or whether the ingredients were loaded into HSWG as fully segregated or pre-blended components. During this dry phase, finer APAP particles percolated through the MCC bed to the bottom of the granulator. The final inhomogeneous API distribution within granule fractions was a result of the combined influences of dry bed segregation and the kinetics of granule coalescence, growth, attrition and mass flux during the HSWG process [86]. Regarding HSWG, a study focusing on the size segregation of fractions of granules instead of component distribution investigated the influence of diluent (lactose vs. mannitol), binder (hydroxypropylmethylcellulose, HPMC vs. povidone, PVP) and the binder addition method (pouring, dripping or spraying). The best combination was identified as mannitol and HPMC due to the lower fine content, when water was added by pouring [87].

An in-depth study into the segregation of lactose and magnesium stearate as model hydrophilic and hydrophobic ingredients during twin-screw wet granulation was carried out by Mundozah et al. The demixing potential in each section of the barrel was researched in detail and several complex segregation phenomena were detected by means of NIR chemical imaging at different locations. For instance, in the wetting zone, the incorporation of the hydrophobic component depended on the extent of the preferential wetting of lactose, which was affected by the binder liquid viscosity. On the other hand, in further barrel sections, the component distribution underwent changes due to particle collisions, abrasion and chipping, and at the final stage the granules regained their homogeneity owing to competing breakage and consolidation mechanisms. In summary, the authors proposed to map demixing potential as a function of a dimensionless mixing number, which is described by mean residence time, torque and the free volume of the twin-screw granulator [88].

### 4.3. New Perspective: Continous Manufacturing for the Improvement of Content Uniformity

**Figure 10:**
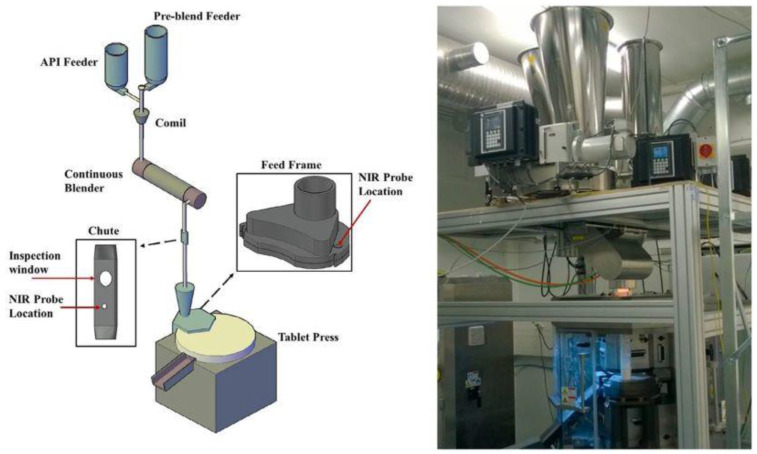
(**Left**): A schematic of a continuous direct compression (CDC) line, adapted with permission from [90]; published by Elsevier, 2019. (**Right**): An overview of a CDC line setup, adapted with permission from [21]; published by Elsevier, 2019.

Finally, attention must be given to the rise of continuous manufacturing in the context of ensuring drug product uniformity and other CQAs. Among the numerous advantages of this approach for current pharmaceutical industry practice [89], the potential for mitigating blend segregation throughout reduced unit operation steps and for improving the CU of solid dosage forms cannot be overlooked, as proven by several studies on continuous direct compression (CDC, Figure 10).

Oka et al. employed five model blends at different concentrations and of different sifting segregation potentials, as determined with ASTM tests, in order to compare their homogeneity and segregation behavior between a batch V-blender and a continuous tubular blender (by assessing component RSD at different locations or time points). The authors also developed a simple mathematical model correlating a blend’s segregation index to a dimensionless ‘material property metric’, which is governed by the ingredients’ d_50_ value and density. Again, it was confirmed that free-flowing materials exhibit a higher segregation tendency and the relationship was described by a quadratic function, whereas for poorly flowing blends the segregation extent was limited. With the exception of one model mixture (MCC and copper sulfate), the batch mixer resulted in much worse uniformity of blends susceptible to segregation and a linear correlation was revealed between the mixtures’ segregation index and RSD value. On the contrary, such a relationship was absent when a continuous mixer was used, as segregation did not occur. This is due to different trajectories in these types of equipment. In a tumbling blender, large particles are characterized by higher inertia than fines of similar density, whereas particles of similar size but higher density tend to sink to the bottom. On the other hand, in a continuous mixer the particles’ movement space is restricted and their trajectory is determined by processing conditions. As a result, segregation is inhibited by proper residence time and impeller speed, which is dependent on the blades’ configuration and the mass flow rate. In summary, continuous mixing was proven to be effective in ensuring homogeneity, even for blends that were susceptible to segregation and failing uniformity in batch processes [91].

In another study, the design of experiments approach was used to evaluate the influence of continuous-mixing conditions on the CQAs of tablets directly compressed from a blend containing low-dose, cohesive APIs. Two API doses, total mass flow rates (11–42 kg/h), mixer speeds (125–175 rpm) and mixer configurations (22.5°-angled paddles alternating between forward- and backward-facing vs. 45°, all forward-facing) were investigated. It was found that for the studied blend the configuration of mixing elements and the mixer speed did not influence tablets’ content uniformity. At low mass flow rates, the initial API content was lowered relative to the label claim due to the adsorption of the material characterized by a high surface area to the equipment interface. Nevertheless, Bayesian analysis demonstrated that regardless of the studied settings, the CU acceptance criteria would be met with 95% likelihood, which proved the robustness of the continuous mixing process for low-dose, cohesive APIs [92]. A study by Sierra-Vega et al. compared the effect of continuous mixing speed in the range of 106–496 rpm (target value of 250 rpm) on content uniformity, monitored using NIR at three stages: in the feeding chute (I.D. 3 in.) connecting the continuous blender to the tablet press, in the feed frame (Fill-O-Matic type) and in tablets. It was found that with extreme mixing settings, the API concentration variability in the chute was higher, with a mean value about 4% lower than the target value, but in the feed frame the API content was consistently within 90–110% of the target regardless of the mixing speed. Effectively, the tablets’ CU was acceptable in all cases. The results demonstrated that segregation during the blend transfer in CDC does not have to translate into poor uniformity of the final product, and early segregation events may be compensated for at the later stages of the process, as assured by the mixing effect in the feed frame [90].

In another group of studies, the robustness of a CDC line (Figure 10, right) without additional conveyors was tested with blends that were prone to segregation and uniformity losses in batch processing (with large particle size and density differences) [21,93,94]. In the first design-of-experiments investigation, the following independent variables were explored with respect to their influence on tablet CU as a response—API grade (standard or granular acetaminophen), mannitol grade, API load (2–22%), total feed rate (3.5–7.5 kg/h) and continuous mixer speed (500–1200 rpm). It was revealed that the two grades of API required the building of separate models describing the relationships between variables, as the three-way interaction between API grade, mannitol grade and drug load was significant. As a result, various parameter combinations were mapped to identify the optimal and suboptimal settings for acceptable content uniformity. In brief, it was confirmed that the base scenario of the CDC process (22% of standard-grade acetaminophen with a small mannitol grade) had the lowest segregation risk. The effect of the feed rate and mixing speed was dependent on the combination of the formulation variables. In general, it was found that the highest segregation probability and CU loss concerned the combinations of a high load of granular API or a low drug load of a standard API grade with large filler particles [93]. The same formulations were further evaluated in sifting and fluidization ASTM tests and compared between batch and continuous direct compression. The highest segregation index was determined for 22% granulated API with small-sized mannitol, the lowest was observed for the formulations containing standard grades of both active and filler ingredients. In general, the mean acetaminophen content in tablets was similar between batch and continuous compression methods. However, variability was improved with lower RSD for CDC, probably because the batch process involved gravitational feeding from an IBC to the tablet press. The only exception in which traditional tableting was superior concerned a highly cohesive formulation which did not segregate, but stuck to the funnel at the continuous mixer entry, which highlights the necessity of design optimization for every element of a CDC line. It is also interesting to note the fact that, contrary to expectations, the segregation indices determined in the sifting and fluidization tests did not translate directly to practical content uniformity results in tablets [21]. The final study of the aforementioned formulations explored the effect of different feeding rates of ingredients at the beginning of the line before the mixing step, as well as disturbances imitating the feeder refilling or the sticking and detaching of the API to or from equipment surfaces. Again, granular acetaminophen displayed more baseline variability, whereas stable standard API formulations were more sensitive to disturbances, although they still passed the CU criteria, while the former did not. Tolerance for feed and flow rate changes was calculated to determine to which extent fluctuations would be acceptable without API content in tablets deviating from ±25% of the label claim. Additionally, it was confirmed that due to the broader residence time distribution, the feed frame was capable of backmixing and smoothing these flow disturbances—even to a higher degree than the use of a continuous blender as the main mixing element [94].

## 5. Conclusions

Solid dosage forms may experience issues with drug content uniformity or sub- or superpotency due to the segregation of initially well-mixed blends. This demixing may occur according to sifting, fluidization or trajectory (rolling) mechanisms, and the primary driving force behind this phenomenon is the difference in particle size between ingredients. Moreover, other blend properties, such as the fines concentration, density or flow, may play a part, and whether segregation is initiated depends heavily on the equipment used (the geometrical design, dimensions and friction) and the processing conditions, such as vibrational stresses or shearing related to blend mixing. Regarding the manufacturing of tablets, the risk of segregation exists at several stages of the blend’s fate: the transfer from bulk containers to tablet presses, feeding from the press’s hopper or during the filling of dies in both eccentric and rotary machines. Moreover, the possibility of demixing is involved not only in direct compression, but may also concern granulation processes.

Although the sifting and fluidization segregation potential of pharmaceutical blends may be evaluated by means of various types of laboratory-scale tests, the results may or may not correlate to practical issues with the content uniformity of tablets in industrial-scale manufacturing. Computational tools such as DEM have also helped in the mechanistic understanding of flow behavior at different stages of the manufacturing process. It must be stressed that the occurrence of axial and radial segregation and its impact on real drug homogeneity is a complex phenomenon, depending on the interplay between an array of factors—the API load in the blend, its particle size and density ratio to excipients, blend cohesivity, friction properties, mixing conditions and the residence time distributions at the consecutive stages of the blend’s route, the geometry of the equipment elements governing the powder flow, etc. Moreover, segregation may occur at one stage of the process (e.g., the feeding chute), but be counteracted by backmixing at another (e.g., in the feed frame). Therefore, these formulation and processing variables should not be considered in isolation; instead, a thorough investigation of the given conditions and their impact on CU is recommended, especially for low-dose, highly potent tablets.

The current review of pharmaceutically relevant studies on blend segregation and content uniformity seems to reinforce the general basic recommendations for the reduction of demixing, derived from other engineering areas. For instance, when considering the discharge from an IBC or feeding hopper, their design should ensure a mass flow instead of a funnel flow pattern, probably via a conical geometry, steep wall angles or appropriate bin inserts. Wide pipe diameters, minimal drop heights or alternative venting systems may reduce fluidization during the gravitational transfer of blends. During die filling, the flow pattern will depend on the blend’s properties, and the influence of the feed frame and turret speed should be considered on a case-by-case basis for the potential impact of particles’ residence time on induced segregation or, on the contrary, on the occurrence of additional mixing steps. In general, the suction conditions during the die filling produce better uniformity than gravity filling.

Apart from equipment design and operating parameters, care must be taken to optimize the choice of a formulation and process. Granulation, especially roller compaction, may be a way to improve content uniformity. Regarding direct compression, preformulation studies are vital for the optimization of blend composition. Although particle morphology may play a part in the propensity for segregation, major consideration must be taken to diminish particle size ratios between APIs and excipients. Since cohesive materials are less susceptible to demixing, a blend’s flow properties should ideally be balanced via the choice of excipients to assure smooth handling and transfer without excessive segregation. Many examples have indicated that a high load of finer ingredients or fractions is beneficial for the reduction of demixing, although increasing the drug load may in fact result in the opposite effect. Therefore, every formulation and process should be carefully considered to determine the optimal range and robustness, and segregation risk mapping may be a useful tool to guide this development process. Finally, it must be stressed that a major perspective for uniformity improvements is offered by continuous manufacturing, which has successfully demonstrated both robustness and reduced segregation events when compared to traditional batch processing.

## Data Availability

Data sharing not applicable.

## Appendix A

The literature search was carried out using PubMed (PM) and Academic Search Ultimate (ASU) databases for the year range 2005–2021. The terms used and the number of items retrieved were “blend segregation” (355 for PM and five for ASU, respectively), “powder segregation” (236 PM and 25 ASU), “content uniformity AND tablets” (544 PM and 329 ASU). The records were screened according to the titles and abstracts for the following inclusion criteria: (1) original research articles; (2) English language; (3) published in Impact Factor journals; (4) studies employing pharmaceutically relevant blends or equipment; (5) application to direct compression blends or granulation blends for the actual or potential production of monolithic tablets; (6) description of relationships between equipment, processing or formulation parameters to segregation events and/or tablet content uniformity. Consequently, reports on other types of dosage forms, works focused solely on the mixing process and initial blend uniformity, accounts of analytical method development or simple descriptions of product development were excluded as being outside of the scope of the review. Guided by these criteria and excluding duplicates, 38 articles were found to be eligible and were included in the review. Furthermore, relevant studies not found in the search results but referenced in the reviewed literature were also incorporated.
